# Effect of Ball-Milling Pretreatment of Cellulose on Its Photoreforming for H_2_ Production

**DOI:** 10.1021/acssuschemeng.1c07301

**Published:** 2022-04-04

**Authors:** Lan Lan, Huanhao Chen, Daniel Lee, Shaojun Xu, Nathan Skillen, Aleksander Tedstone, Peter Robertson, Arthur Garforth, Helen Daly, Christopher Hardacre, Xiaolei Fan

**Affiliations:** † Department of Chemical Engineering, School of Engineering, The University of Manchester, Manchester M13 9PL, United Kingdom; ‡ State Key Laboratory of Materials-Oriented Chemical Engineering, College of Chemical Engineering, Nanjing Tech University, Nanjing 210009, P. R. China; § UK Catalysis Hub, Research Complex at Harwell, Didcot OX11 0FA, United Kingdom; ∥ Cardiff Catalysis Institute, School of Chemistry, Cardiff University, Cardiff CF10 3AT, United Kingdom; ⊥ School of Chemistry and Chemical Engineering, Queens University Belfast, Belfast BT9 5AG, United Kingdom

**Keywords:** Cellulose, Photoreforming, H_2_ production, Ball-milling, Recrystallization

## Abstract

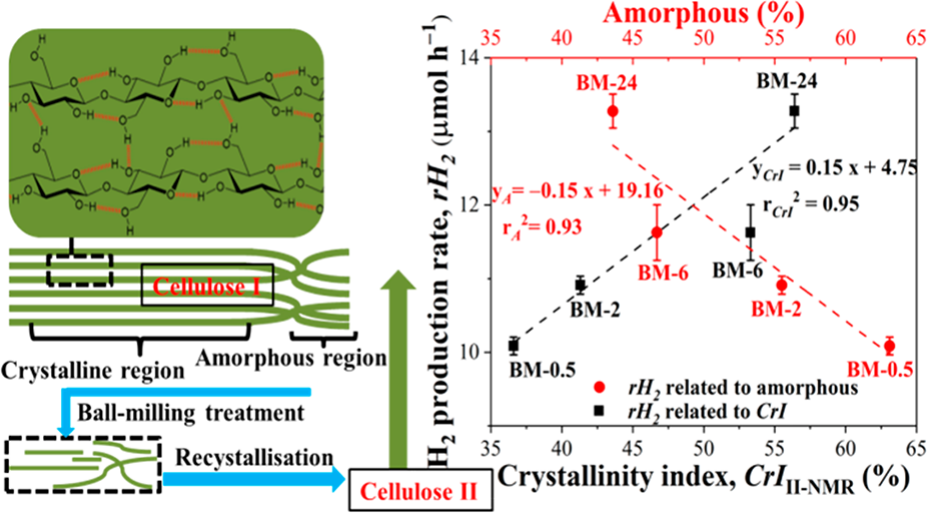

Photoreforming of cellulose is a promising route for sustainable H_2_ production. Herein, ball-milling (BM, with varied treatment times of 0.5–24 h) was employed to pretreat microcrystalline cellulose (MCC) to improve its activity in photoreforming over a Pt/TiO_2_ catalyst. It was found that BM treatment reduced the particle size, crystallinity index (*CrI*), and degree of polymerization (*DP*) of MCC significantly, as well as produced amorphous celluloses (with >2 h treatment time). Amorphous cellulose water-induced recrystallization to cellulose II (as evidenced by X-ray diffraction (XRD) and solid-state NMR analysis) was observed in aqueous media. Findings of the work showed that the BM treatment was a simple and effective pretreatment strategy to improve photoreforming of MCC for H_2_ production, mainly due to the decreased particle size and, specifically in aqueous media, the formation of the cellulose II phase from the recrystallization of amorphous cellulose, the extent of which correlates well with the activity in photoreforming.

## Abstract 2

Ball-milling treatment promoted H_2_ production in cellulose photoreforming, and this process has the potential to produce clean energy (H_2_) sustainably.

## Introduction

To reduce the dependence of the energy supply on fossil fuels, which are unsustainable and associated with greenhouse gas emissions, a transition to renewable energies, such as solar energy, biomass, and hydrogen (H_2_), is necessary.^1−3^ Among the renewable energy sources, sunlight is regarded as an inexhaustible source for sustainable use.^4^ Through photocatalytic reactions, light can be used to convert chemical feedstocks and water into fuels such as H_2_, CH_4_, and alcohols.^5^ Cellulose is one of the most abundant renewable carbon sources in nature^6^ and is regarded as an energy source with the potential to contribute to sustainable future energy demands, providing it can be converted efficiently to fuels.^7^ Energy crops,^8−10^ such as *Miscanthus* (40–60% cellulose^11^) and wheat straw (29–45% cellulose^12^), and cellulosic materials,^13−15^ such as waste paper and wood, are possible sustainable resources. Photocatalytic reforming (or photoreforming) is one of the sustainable routes for green production of H_2_,^16−19^ in which photoexcited semiconductors are used to drive reforming reactions of cellulosic resources under ambient conditions.

Cellulose is composed of crystalline and amorphous regions, and the proportion of the two varies, depending on the source of cellulose.^20^ The amorphous structure of cellulose is intrinsically less ordered compared to the crystalline structure, which facilitates interaction with a reactant.^21^ Crystalline cellulose consists of cellulose chains (as shown in Figure S1 in the Supporting Information, SI), in which alternating glucose units are connected in opposite directions by β-1,4-glycosidic bonds with hydrogen bonds existing between O_3_···O_5_ and O_2_···O_6_ hydroxyl groups.^7^ Accordingly, due to the structural features of crystalline cellulose, the direct conversion of cellulose is challenging. Hydrolysis is normally required to deconstruct crystalline cellulose by cleaving β-1,4-glycosidic bonds; however, the accessibility of β-1,4-glycosidic bonds is still hindered by the hydrogen bonding between cellulose chains.^7,22^ Therefore, breaking the hydrogen bonds in the cellulose structure is normally considered one of the key factors to increase the rate of cellulose hydrolysis, which can be achieved by pretreatments.

Pretreatments aimed at modifying the cellulose structure can be classified primarily into three categories: physical, chemical, and biological treatments. Biological treatments use enzymes to hydrolyze cellulose, which is environmentally friendly^23,24^ but lengthy (to achieve considerable conversion^12^). For example, biological conversion of cellulose reducing sugars required 48 h to reach a 28% conversion in a batch system.^25^ Chemical treatments, such as mercerization^26−28^ and ionic liquid (IL)^29−31^ treatment, can also change the structure of cellulose. For example, the degree of polymerization (*DP*) of cellulose was found to decrease after IL treatment using *N*-methylmorpholine-*N*-oxide monohydrate (NMMO-MH)^29^ and 1-butyl-3-methylimidazolium chloride (BMIMCl)^30^ on cellulose pulp (by 6.5%, for 125 min)^29^ and wheat straw cellulose (by 45%, for 10 min).^30^ In addition, treatment of cellulose I with 5 M NaOH and washing with water generated cellulose II hydrate, which on drying formed cellulose II. The enzymatic hydrolysis activity of cellulose to glucose was higher for cellulose II (74%) than for cellulose I (55%) after 24 h of reaction.^32^ Chemical pretreatments requiring a short reaction time are relatively quick but can be relatively complex with associated chemical wastes, which is not ideal.^12^ Ball-milling (BM) is a common physical treatment to amorphize (*i.e.*, reduction in *DP* and crystallinity (*CrI*)) and reduce the particle size of cellulose and has previously been used as a pretreatment for cellulose hydrolysis to glucose.^33−35^ For example, Ribeiro et al. found that the *CrI* of microcrystalline cellulose (MCC) decreased from 92 to 33% after BM at 10 Hz for 48 h.^35^ A decrease in the *DP* of MCC was also noted after BM but was not as significant as the decrease in the *CrI*.^22,35^ For example, the *DP* of MCC decreased from 221 to 191 after BM (at 10 Hz for 48 h), while the *CrI* decreased from 92 to 33%.^35^ In addition to the changes in the *DP* and *CrI* of MCC after BM, the treatment could also significantly reduce the size of the particle.^35^

Significant depolymerization of cellulose (*DP* < 7) has been performed by BM in the presence of acids (H_2_SO_4_/HCl)^36,37^ or solid acid catalysts^38^ (*i.e.*, kaolinite), producing soluble oligosaccharides. Within these studies the energy efficiency/sustainability has been assessed for different milling techniques (*i.e.*, attritor versus mixer mill), with the attritor mill showing reduced energy consumption at a larger scale, *i.e.*, 100 kg showed a 46-fold decrease in energy consumption compared to milling at a 1 kg scale.^38^ Enhanced energy efficiency of acid-catalyzed cellulose depolymerization at a larger scale was also demonstrated in the study of Rinaldi and co-workers, wherein the energy consumption was assessed with a significant decrease from ca. 200 to 9.6 MWh·t^–1^ when milling at a 1 kg scale compared to a 1 g scale (planetary ball mill). These energy efficiencies and the potential reduction in the environmental impact^39−41^ of BM make feasible its use as a sustainable pretreatment method for cellulose.

BM has been widely used as a pretreatment of cellulose for hydrolysis reactions to form glucose, e.g., the glucose yield (of the BM-treated cellulose) reached 60–90% in enzymatic/hot-compressed water hydrolysis.^22,33,42^ Although the effect of cellulose pretreatments on hydrolysis/enzymatic hydrolysis has been well-studied, relevant studies on H_2_ production from photoreforming are rather limited.^43^ The amorphous portion of the BM-treated cellulose was believed to improve the hydrolysis of the treated cellulose, which was regarded as the first step of the photoreforming of cellulose. However, it is worth noting that other research also showed the important effect of the cellulose structure for photoreforming activity in an aqueous system. Chang et al.^44^ reported a 2-fold increase in the H_2_ production rate (*r*H_2_) from the photoreforming of tetrabutylammonium hydroxide (TBAH)-treated cellulose, which was attributed to the crystal transformation of cellulose I to cellulose II during this chemical pretreatment. The decreased crystallinity and rearranged H-bonding network of cellulose II over cellulose I was assumed to be key to increasing the interaction between the catalyst and cellulose and, hence, improving the *r*H_2_. Therefore, the effect of the BM treatment of cellulose on its production of H_2_ requires further investigation, including understanding the impact of the structural changes of cellulose on the photoreforming activity.

Herein, the effect of the variation in structural properties of ball-milled MCCs on their activity in photoreforming was investigated. Water-promoted recrystallization of amorphous cellulose during the photoreforming reaction was characterized, and the recrystallization property of BM-treated cellulose was then correlated with its activity with respect to H_2_ production. The findings revealed a good correlation between recrystallization and the improved activity of H_2_ generation of BM-treated MCCs in photoreforming reactions. This work investigated the mechanism of photoreforming BM-treated cellulose for H_2_ production and enabled the establishment of the relevant property–activity relationship.

## Experimental Section

### Chemicals and Materials

Microcrystalline cellulose (MCC, Aldrich) with a density of 0.6 g cm^–3^ was used in this work to be pretreated by ball-milling (BM). The chemicals (*i.e.*, phenol, sulfuric acid, disodium 2,2′-bicinchoninate, Na_2_CO_3_, NaHCO_3_, CuSO_4_·5H_2_O, and l-serine) used to prepare the phenol acid solution and 2,2′-bicinchoninate (BCA) solution were purchased from Sigma-Aldrich. Standard solutions (including cellobiose, glucose, d-galactose, glyceraldehyde, and formic acid) and mobile phase (5 mM sulfuric acid solution) for high-performance liquid chromatography (HPLC) analysis were all purchased from Sigma-Aldrich. Deionized water was obtained from a Direct-Q 3UV ultrapure water system (Millipore).

### Pretreatments of MCC

BM of the MCC was conducted using a laboratory ball-mill (Retsch PM100 planetary ball-mill). Three g of the pristine MCC was placed in the grinding jar (volume = 50 mL, depth = 3.5 cm, and diameter = 4.2 cm) together with 10 ZrO_2_ ceramic balls (*d* = 1 cm), and the ball-mill was operated at 500 rpm for various grinding durations of 0.5, 2, 6, 16, and 24 h. The resulting treated MCCs were denoted as BM-*x* (*x* = 0.5, 2, 6, 16, and 24, respectively).

### Characterization of Materials

The crystallinities of the pristine and treated MCCs were characterized by powder X-ray diffraction (XRD). XRD patterns of the MCCs were obtained with a PANalytical X’Pert Pro X-ray diffractometer using Cu Kα emission lines (λ = 1.5406 Å, at 40 kV and 40 mA). XRD scanning was performed at 2θ from 10° to 90° with a step size of 0.033°. The degree of crystallinity of the materials was represented by the crystallinity index (CrI), which was calculated using Segal’s method according to eqs 1 and 2.^44,45^
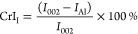
1
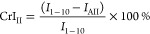
2where *I*_002_ and *I*_1–10_ represent the maximum intensities of the (002) and (1–10) lattice diffraction of cellulose I and cellulose II (2θ = 22.5° and 19.8°), respectively, and *I*_AI_ and *I*_AII_ represent the intensities of diffraction for the amorphous phase in cellulose I and cellulose II at 2θ = 18° and 16°, respectively.

The morphology of the MCCs was characterized by scanning electron microscopy (SEM, Joel JSM-6610LV at 20 kV, 20 nm Au coating for all samples), and the particle-size distribution was obtained by manually measuring the size of ∼200 particles of each sample using ImageJ.

Attenuated total internal reflection infrared spectroscopy (ATR-IR) of the MCCs was performed using a Bruker Vertex 70 spectrometer with a deuterated triglycine sulfate (DTGS) detector and a platinum ATR accessory (diamond crystal). The background spectrum of the crystal and spectra of the treated MCCs were recorded with 64 scans at a resolution of 4 cm^–1^.

The number-average degree of polymerization (*DP*) of the MCCs was estimated by comparing the ratio of the glucosyl monomer concentration (*C*_GM_) to the reducing-end concentration (*C*_RE_), as shown in eq 3.^46^ The concentrations of the two were determined by the phenol–sulfuric acid method and BCA method, respectively, as described elsewhere.^46,47^
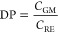
3

The absorbance of visible light (490 and 560 nm) of the phenol–sulfuric acid solution and BCA solution with MCC or glucose was obtained by UV–visible diffuse reflectance spectroscopy (UV–vis DRS, Shimadzu UV-2600) to determine *C*_GM_ and *C*_RE_ of the MCCs, respectively. For UV–vis DRS analysis, glucose solutions with different concentrations were prepared to establish the calibration curves for quantitative determination of *C*_GM_ and *C*_RE_ values of the MCCs. Glucose is the shortest and simplest unit in cellulose, and it has only one reducing end; therefore, the values of *C*_GM_ and *C*_RE_ in a glucose solution equal the concentration of glucose.^46^

{^1^H-}^13^C cross-polarization (CP) magic angle spinning (MAS) nuclear magnetic resonance (NMR) spectra were recorded using a Bruker 9.4 T (400 MHz ^1^H Larmor frequency) AVANCE III spectrometer equipped with a 4 mm HFX MAS probe. Experiments were acquired at ambient temperature using a MAS frequency of 10 kHz. The ^1^H π-pulse duration was 5 μs, the ^13^C π-pulse duration was 20 μs, and ^13^C spin-locking at ∼25 kHz was applied for 2 ms, with corresponding ramped (70–100%) ^1^H spin-locking at ∼50 kHz for CP experiments with 100 kHz of SPINAL-64^48^ heteronuclear ^1^H decoupling used throughout ∼30 ms of signal acquisition (with 12.6 μs dwell time between complex data points). A CP contact time of 2 ms has been shown to minimize errors in cellulose crystallinity analysis.^49^ A Hahn-echo τ_r_–π–τ_r_ sequence of 2 rotor periods total duration was applied to ^13^C after CP to circumvent receiver dead time. Samples were treated and packed into 4 mm (outside diameter, o.d.) zirconia rotors and sealed with a Kel-F rotor cap. Between 1 024 and 16 384 transients were coadded for each sample, with repetition delays set to 1.3^1H^*T*_1_. Spectral deconvolution and peak fitting were performed in the solid line shape analysis (SOLA) module v2.2.4 in Bruker TopSpin v4.0.9. The ^13^C peak deconvolution and assignment was based on the work by Idström et al.^50^ and Larsson et al.^51^

The *CrI*_I-NMR_ (or *CrI*_II-NMR_) was calculated according to the deconvolution and eq 4
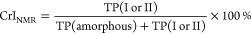
4where *CrI_NMR_* is the crystallinity index of cellulose I (or II) calculated from ^13^C ssNMR spectra, TP (I or II) and TP (amorphous) are the total proportion of the deconvoluted peaks assigned to crystalline cellulose I (or II) and amorphous cellulose from the C4 or C6 regions in the ^13^C ssNMR spectra, respectively.

### Effect of Water Exposure for the BM-Treated MCCs

MCC-0 and the ball-milled MCCs were dispersed in water (stirred at 40 °C for 30 min), and the treated MCCs were recovered by centrifugation and dried (at 60 °C for 12 h) before characterization by XRD, ATR-IR, and ssNMR.

### Photoreforming of MCCs

Photoreforming of the MCCs was performed in a photoreactor, which has been described in detail elsewhere.^52^ The catalyst used was Pt (0.16% theoretical loading) supported on m-TiO_2_ (a TiO_2_ mixture of 85 wt % anatase and 15 wt % rutile), denoted as 0.16%-Pt/m-TiO_2_, which was prepared by impregnation (as described in the Supporting Information).^52^ The procedure for the photoreforming experiments was as follows: 75 mg of 0.16%-Pt/m-TiO_2_ and 100 mg of MCC were placed in the reactor, to which 100 mL of deionized water was then added. The system was stirred thoroughly for 0.5 h at room temperature and purged with argon (Ar) for 1.5 h to remove the dissolved oxygen from the mixture. The reactor was then sealed and irradiated by a UV-A lamp (365 nm, 2 × 8 W, Thistle Scientific) for 5 h at 40 °C. During the catalysis under UV irradiation, a hydrogen (H_2_) microsensor (H2-NPLR needle sensor, Unisense) was used to measure the H_2_ concentration in the headspace of the reactor. The initial and final gaseous products from the headspace of the reactor were also sampled and analyzed by gas chromatography (GC, PerkinElmer Clarus 580 GC, fitted with two 2 m inline HayeSep DB 100/120 mesh columns followed by a 2 m ShinCarbon ST 100/120 mesh column equipped with a thermal conductivity detector (TCD) and a flame ionization (FID) detector). The average production rate of gases (*r*M) produced over 5 h of irradiation was defined as the moles of gas (M) generated per hour (μmol h^–1^).

The efficiency of the radiative energy of the system was determined by the apparent quantum yield (*Φ*_a_), as defined by eq 5.
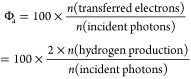
5where *n* (transferred electrons) was determined by the H_2_ production according to the H_2_ generation reaction (eq 6) during the photoreforming and *n* (incident photons) was measured by a potassium ferrioxalate actinometer, which was described by Bolton et al.^53^
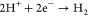
6

### Determination of Soluble Compounds from MCC Pretreatments

Fifty mg of MCC-0 and the BM-treated MCCs were washed in 2.5 mL of deionized water by shaking the suspension for 5 min. The suspension was then filtered to obtain the filtrate. The filtrates (10 μL) were then analyzed using an Agilent 1260 infinity HPLC system equipped with a refractive index detector (HPLC-RI) and a Rezex ROA-organic acid H+ column (300 × 7.8 mm) to determine if soluble compounds (such as glucose and cellobiose) were present in the filtrate as a result of the pretreatment. The flow rate of the isocratic mobile phase (5 mM H_2_SO_4_) was set at 0.5 mL min^–1^, and the RI and column temperatures were both 40 °C. HPLC profiles of the various commercial standards (including oligo-/monosaccharides and a range of sugar oxidation products) were obtained, and a calibration curve was established for quantitative analysis.

## Results and Discussion

### Ball-Milling Treatment of MCC

Comparative photoreforming performances (as a function of time-on-stream, ToS) of MCC-0 and BM-treated MCCs are shown in Figure S2. All the BM-treated MCCs showed an improved performance toward H_2_ production when compared to MCC-0. For example, under UV-A irradiation for 0.5 h, BM-0.5 produced 50.4 μmol of H_2_, which was 25.4% higher than that produced by the system with MCC-0 (∼40.2 μmol). The data demonstrate that prolonged BM treatment was beneficial to H_2_ production from photoreforming, with BM-24 producing 65 μmol of H_2_. To understand the effect of BM on the properties of the resulting MCCs and their photoreforming performances, comprehensive characterization of the BM-treated MCCs was carried out.

BM as a means of decrystallizing cellulose has been well-established,^54−56^ and changes in crystallinity (*CrI*) and *DP* have been observed after BM, which have resulted in improved activity.^54,55^ In this work, the *DP* of the BM-treated MCCs was determined to study the relationship between *DP* and *r*H_2_ (and Φ_a_). Table 1 shows that an increase in the duration of BM reduced the *DP* of the resulting MCCs. The findings from the BM-treated MCCs show that the *DP* of the MCCs did not correlate with their photoreforming activity strongly (Figure S3). For example, the values of *r*H_2_ and *Φ*_a_ for BM-0.5 (*DP* = 165.4) were 10.1 μmol h^–1^ and 32.5%, respectively, while the values for BM-24 (*DP* = 32.1) were only slightly increased to 13.3 μmol h^–1^ and 42.8%, respectively.

**Table 1:** Properties and Average H_2_ Production Rate (*r*H_2_) of MCC-0 and the BM-Treated MCCs

sample	BM time (h)	*CrI*_I_^a^ (%)	particle size (μm)	*DP*	*r*H_2_ (μmol h^–1^)
MCC-0	0	81.3	30–240	162.0	8.0
BM-0.5	0.5	24.7		165.4	10.1
BM-2	2	2.4	4–20	78.7	10.9
BM-6	6	0		52.3	11.6
BM-16	16	0	4–20	31.4	12.7
BM-24	24	0	4–20	32.1	13.3

a Crystalline index was calculated from the XRD results using eq 1.

BM was also observed to alter the particle size and crystallinity of the MCC significantly, as shown in Figures S4 and 1a. SEM analysis of the MCCs (Figure S4) showed that the BM treatment reduced the particle size of the MCC significantly from 30–240 μm (for MCC-0) to 4–20 μm after 2 h BM. A further increase in the BM treatment time did not bring significant changes to the morphology and particle sizes of the treated MCCs, as evidenced by Figures S4e–h. A slight color change of the MCC (from white to light yellow), however, was observed after the BM treatment for extended milling time (24 h), which suggests the possible decomposition of MCC to, for example, furanic derivatives or humins.^34^

**Figure 1:**
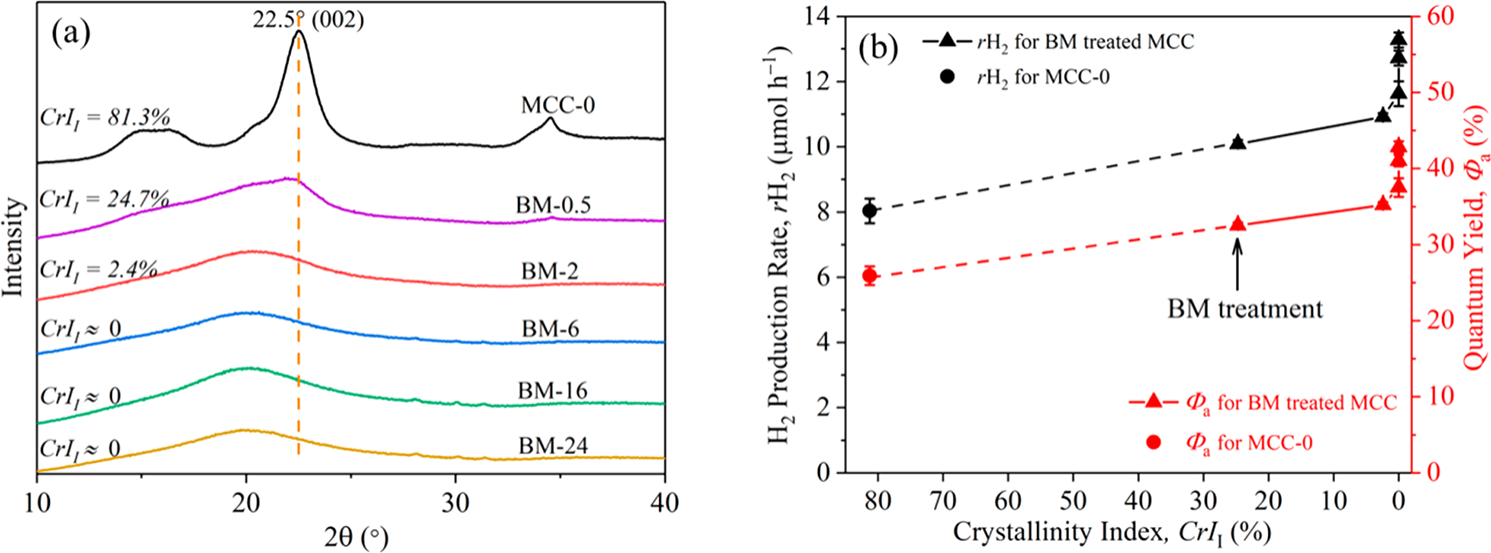
(a) XRD spectra of MCC-0 and the BM-treated MCCs and (b) average H_2_ production rate (*r*H_2_, black axis) and quantum yield (red axis) of the photocatalytic reactions against the crystallinity of BM-treated MCC. XRD measurements of the samples were done directly after milling.

CrI values of the BM-treated MCCs (Table 1) reduced significantly after 2 h BM to become nearly fully amorphous. Figure 1a shows that MCC-0 had a strong peak at 2θ = 22.5°, which was assigned to the cellulose I crystalline plane (002).^33^ The *CrI*_I_ value of MCC-0 was 81.3%, while that of BM-2 was only 2.4%, which demonstrated the effectiveness of BM for decrystallizing the MCC (note: the *CrI*_I_ of 0% suggested completely amorphous MCCs, *i.e.*, BM-6, BM-16, and BM-24, as evidenced by XRD; Figure 1a). The crystalline structure of cellulose contained ordered cellulose chains that were held by hydrogen bonds.^57^ The mechanical forces (*i.e.*, collision, compressive, and attrition force) created between the milling balls and the wall of the container altered the MCC crystalline structure by breaking the hydrogen bonds between cellulose sheets.^58^ The conversion of cellulose I to amorphous cellulose after BM was also supported by ATR-IR characterization (Figure S5), and the relevant details can be found in the Supporting Information. BM pretreatment of cellulose for 2 h was found to amorphize the cellulose to near completion. Comparatively, in enzymatic treatment for 24 h (*i.e.*, cellulolytic enzyme GC-220 with β-glucosidase Novozyme-188^59^), amorphization of cellulose could only be achieved partially from 31% to 45%. Hence, ball-milling is an effective way to degrade the cellulose structure.

The variation of the *r*H_2_ and *Φ*_a_ values of MCC-0 and the BM-treated MCCs as a function of the *CrI*_I_ values is shown in Figure 1b. An increase was observed in the photoreforming activity with a decrease in the crystallinity of the MCC samples after the BM treatment. The comparison in activity of MCC-0 (MCC-0, CrI_I_ = 81.3%) and amorphous BM-6 (*CrI* = 0%), *r*H_2_ (from 8.0 to 11.6 μmol h^–1^) and *Φ*_a_ (from 25.9 to 37.5%), showed an increase by ∼45%. On the basis of the findings of previous research, the hydrolysis of MCCs was promoted in the presence of amorphous cellulose,^21^ and these increased reaction rates may be due to the improved accessibility of the internal cellulose structure to the reacting species.^33,42^ However, for the amorphous MCCs (*i.e.,* BM-6/-16/-24, *CrI* = 0%), a further increase in the BM treatment time caused further improvements in photoreforming activity (*e.g.*, *r*H_2_ = 13.3 μmol h^–1^ and *Φ*_a_ = 42.8% for BM-24), which could not be related to the decreased crystallinity of the amorphous MCCs.

### BM-Treated MCCs Exposed to Water

Photoreforming occurs in aqueous media and, therefore, the reactivity of the MCCs will also be influenced by water-promoted recrystallization of amorphous cellulose, which can occur upon its exposure to both water vapor^60,61^ and liquid water^54,62^ at both room temperature^54,60,61^ and elevated temperatures (*e.g.*, 50–100 °C^54,60^ and 110–150 °C^62^). Furthermore, such processes can occur quickly, *e.g.*, <0.5 h.^60^ The crystalline structure after recrystallization depends mainly on the initial structure of the cellulose and the exposure temperature. It was reported that amorphous or largely amorphous cellulose (with the amorphous form >75%)^63,64^ tended to recrystallize to cellulose II,^54,60,61^ while partially decrystallized cellulose I could recrystallize back to cellulose I^54,61,62^ after water exposure. Additionally, a higher exposure temperature (>80 °C) could promote the formation of cellulose IV.^54,60^ In addition, a previous study investigated the effect of solvents (i.e., acetone, benzene, and ethanol) on recrystallization of ball-milled/amorphous cellulose.^63^ It was found that solvents with lower polarity have an insignificant effect on the recrystallization of amorphous cellulose. Accordingly, recrystallization of the BM-treated MCCs during photoreforming (especially at the initial stage) was likely, which subsequently may have affected the activity. To investigate this, the BM-treated MCCs were immersed in water for 30 min at 40 °C (the same as in the photoreforming reaction), recovered, dried, and characterized using XRD, ssNMR, and ATR-IR.

**Figure 2:**
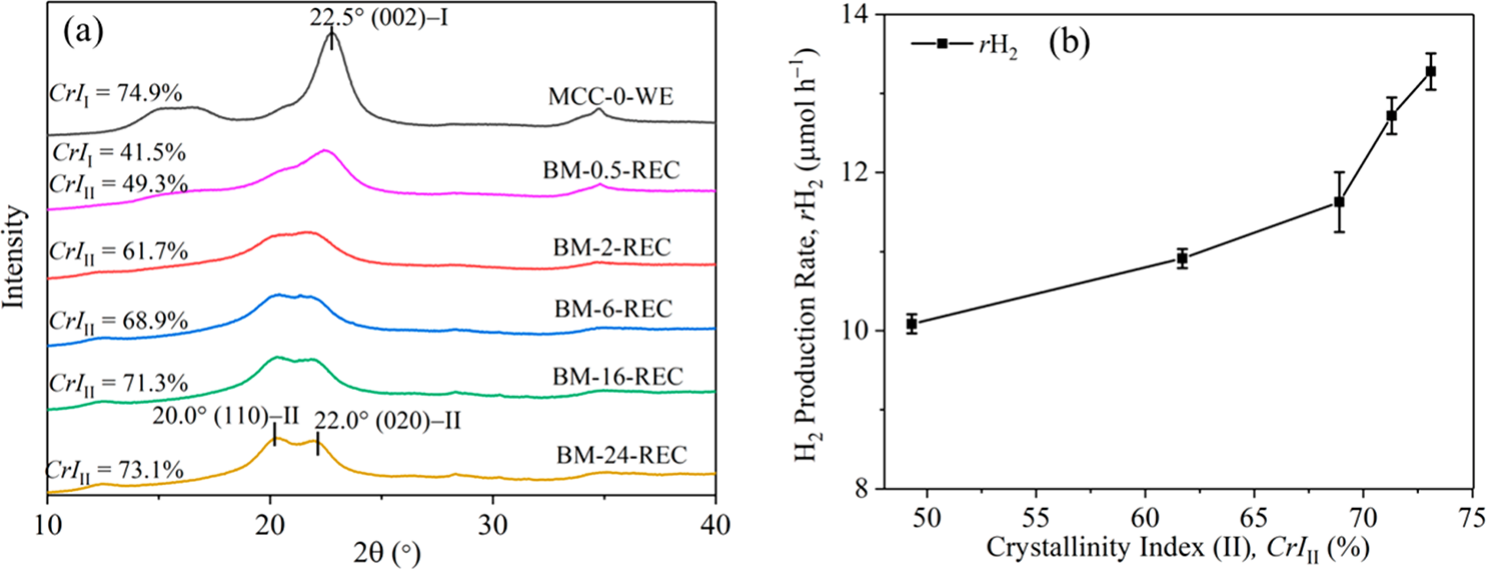
(a) XRD spectra of MCC-0 and the BM-treated MCCs and (b) *r*H_2_ as a function of *CrI*_II_ of the BM-treated MCCs. XRD characterization of the MCCs was done after their exposure to water.

XRD analysis of the water-exposed MCCs (Figure 2a) showed the presence of cellulose I in MCC-0-WE and BM-0.5-REC, as evidenced by the diffraction peak at 2θ = 22.5° (*i.e.*, the crystalline (002) plane of cellulose I). In the diffractograms of BM-0.5-REC, diffraction peaks at 2θ = 20.0° and 22.0°, which represent the (110) and (020) crystalline planes of cellulose II, respectively, were also found.^44^ For the recrystallized BM-treated MCCs, the intensity of the cellulose II peaks increased with an increase in the milling time. The crystallinity indexes of cellulose I (*CrI*_I_) and cellulose II (*CrI*_II_) of the water-exposed MCCs were calculated, as shown in Figure 2a. After the water exposure, the *CrI*_I_ value of BM-0.5 increased from 24.7 to 41.5% (BM-0.5-REC). Partially disrupted cellulose I in cellulose after BM preferred to recrystallize to crystalline cellulose I upon water exposure,^54^ which may be related to the seed nucleation of cellulose I nanocrystals.^64,65^ The cellulose I phase was not detected by XRD for MCC-2/-6/-16/-24 after the water exposure. The formation of cellulose II (likely from the amorphous phase created by BM), however, increased as a function of BM treatment time, *i.e.*, *CrI*_II_ = 49.3% for BM-0.5-REC vs *CrI*_II_ = 73.1% for BM-24-REC. Because recrystallization of the amorphous phase in the BM-treated MCCs to a cellulose II phase was common in water, the amorphous and cellulose II phases in the MCCs could be responsible for the observed increase in activity in the photoreforming reactions.

The *r*H_2_ values of the BM-treated MCCs were plotted against their *CrI*_II_ values (Figure 2b), which showed that *r*H_2_ is correlated with the *CrI*_II_. The *r*H_2_ value increased slightly from 10.1 to 11.6 μmol h^–1^ with a large increase in *CrI*_II_ from 49.3% to 68.9%. Meanwhile, when the *CrI*_II_ increased above 68.9%, the *r*H_2_ increased dramatically by 1.7 μmol h^–1^ with the *CrI*_II_ increasing by only 4.2%. This change at *CrI*_II_ = 68.9% could be due to the overestimation of *CrI*_II_ in BM-0.5-REC and BM-2-REC from the XRD analysis because the diffraction peaks of cellulose I and II in these two samples were difficult to be separated in the XRD analysis. As a result, a large increase of *CrI*_II_ from 49.3% (BM-0.5-REC) to 68.9% (BM-6-REC) could be obtained based on the XRD analysis. In addition, for the recrystallized cellulose, the proportion of cellulose II increased continually according to the XRD analysis; however, XRD only allowed assessment of the crystalline phase rather than changes in the amorphous region. From a previous study, amorphous cellulose has been proposed as the more active component in hydrolysis reactions.^21^ However, as shown in Figure 2b, the *r*H_2_ showed an increased trend with an increase in *CrI*_II_ based on the XRD analysis, which was in contrast with the previous study. Therefore, ssNMR was performed to probe both the crystalline and amorphous cellulose regions in the recrystallized MCCs to further understand the structure–activity relationship of cellulose in photoreforming reactions.

**Figure 3:**
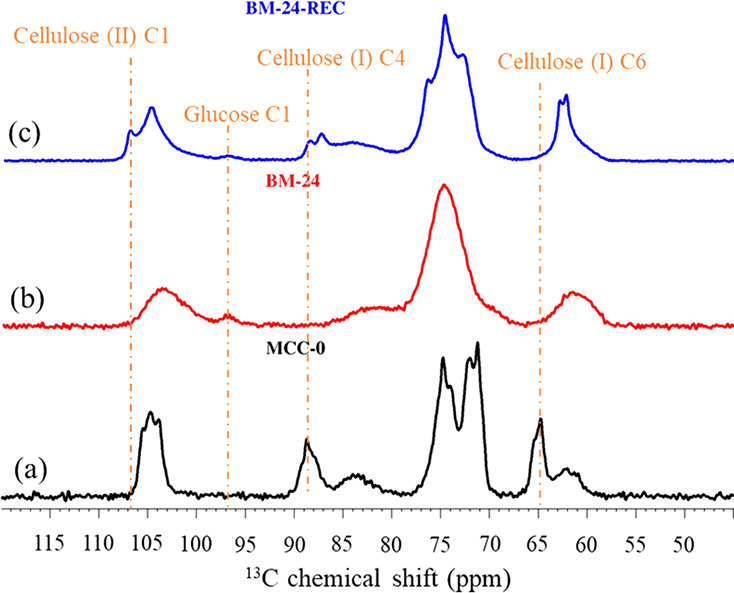
^13^C ssNMR spectra of (a) MCC-0, (b) BM-24 (the MCC after 24 h BM treatment), and (c) BM-24-REC (recrystallized BM-24 after water exposure).

Figure 3 shows the ^13^C magic angle spinning (MAS) ssNMR spectra of MCC-0, BM-24, and BM-24-REC. MCC-0 showed the characteristic ^13^C chemical shifts of crystalline cellulose I at ∼65.8 ppm (cellulose I, C6)^66^ and at ∼89.0 ppm (cellulose I, C4).^67^ After the BM treatment for 24 h, the signals at 65.8 and 89.0 ppm disappeared, with a broad peak emerging at ∼84 ppm that represented disordered cellulose I^67^ (line (b) in Figure 3). This suggested amorphization of crystalline cellulose I due to BM (24 h). After the water exposure of BM-24 (for 30 min), the ^13^C ssNMR spectrum of BM-24-REC showed the characteristic ^13^C chemical shift of crystalline cellulose II at ∼107.2 ppm (cellulose II, C1)^66^ and the absence of the characteristic crystalline cellulose I signal at ∼65.8 ppm, which suggested that the amorphous cellulose in BM-24 recrystallized to cellulose II after water exposure. In addition, a very weak signal at the characteristic ^13^C chemical shift of glucose C1 was observed for BM-24 and BM-24-REC. The findings from ssNMR were in line with the XRD results (Figures 1a and 2a), *i.e.*, recrystallization of the amorphized crystalline cellulose I to crystalline cellulose II upon water exposure.

^13^C ssNMR spectra and crystalline indexes for all of the recrystallized BM-treated MCCs are shown in Figure S6 and Table S1, respectively, and they serve as further evidence to verify the findings from XRD characterization. For all of the water-exposed BM-treated MCCs, the characteristic ^13^C chemical shift of cellulose I (∼65.8 ppm) disappeared and the characteristic signal of cellulose II (∼107.2 ppm) appeared gradually with an increase in the BM treatment time. The C4 region in the cellulose structure showed relatively well-resolved signals compared to the other ^13^C regions (*i.e.*, C1, C2,3,5, and C6) in cellulose, and therefore, the C4 ^13^C chemical shifts were used for cellulose spectral deconvolution (details can be found in the Supporting Information)^66−69^ to determine the crystallinity of cellulose I and/or cellulose II in MCC-0 and regenerated MCCs (the C6 region was also deconvoluted as a reference to confirm the trends). The crystallinity indexes of cellulose I (*CrI*_I-NMR_) and cellulose II (*CrI*_II-NMR_) calculated from the ssNMR measurements for the recrystallized BM-treated MCCs are shown in Table S1. In the C4 region, *CrI*_I-NMR_ was 55.6% for MCC-0, which increased to 57.6% for BM-0.5-REC, while it decreased to 47.8% for BM-2-REC and decreased to 0% for the recrystallized MCC with longer BM treatment (≥6 h). This trend was consistent with the XRD data (vide supra) and also that from the ssNMR of the C6 region; *CrI*_I-NMR_ from C6 showed a similar value for BM-0.5-REC (46.7%), and it decreased with an increase in BM treatment time, eventually to 0% for recrystallized MCCs with longer BM treatment times (≥6 h). However, *CrI*II-NMR in the C4 region increased with longer BM treatment times, *e.g.*, *CrI*II-NMR increased from 36.6 to 56.4% when the BM treatment time increased from 0.5 to 24 h. A similar trend of CrI_II-NMR_ was also observed in the C6 region, from BM-0.5-REC (28.2%) to BM-24-REC (57.5%). Again, this trend was consistent with the XRD data.

For the relative proportion of the amorphous phase, a similar decreasing trend and similar values could be observed between the C4 and C6 regions with an increase in the BM treatment time for recrystallized ball-milled MCCs. This finding again illustrated that an increase in the BM treatment time could increase the proportion of recrystalline cellulose in ball-milled MCCs. The relative proportion of the ^13^C signal of glucose C1 to the total amount of cellulose in the C1 region is also shown in Table S1. The relative proportion of glucose residue of the water-exposed BM samples showed an increasing trend with prolonged BM, *i.e.*, there was 3.8% glucose C1 residue in BM-0.5-REC and 5.5% in BM-6-REC. The proportion of glucose residues reached a plateau with longer BM treatment times (>2 h), which was in line with the *DP* of the BM-treated MCCs (>2 h, i.e., 52.3, 31.4, and 32.1 for BM-6, BM-16, and BM-24, respectively). The NMR results also showed that (i) the *DP* of the BM-treated samples was reduced with the milling time to a minimum (from glucose C1) and (ii) the extended BM led to improved recrystallization.

Recrystallization of the BM-treated MCCs to cellulose II upon water exposure was also evidenced by ATR-IR (Figure S7), and the relevant detailed discussion can be found in the Supporting Information. The changes in the ATR-IR spectra as a function of the BM treatment time on the formation of cellulose II (during recrystallization) were in line with the XRD (Figure 2) and ssNMR (Table S1) analyses.

**Figure 4:**
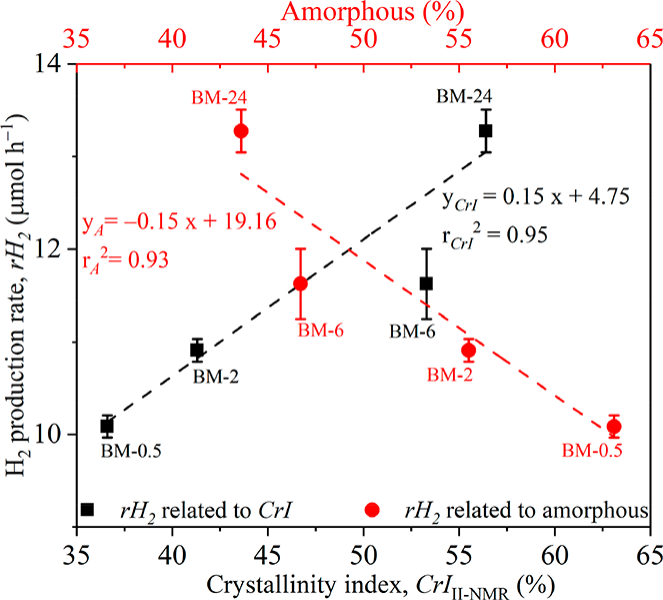
Correlations of *r*H_2_ with the crystallinity index of cellulose II (*CrI*_II-NMR_, bottom *x* axis) and the amorphous composition (top *x* axis) calculated from the C4 region of the recrystallized BM-treated MCCs. Red symbols, correlation of amorphous composition and *r*H_2_; black symbols, correlation of *CrI*_II-NMR_ and *r*H_2_.

To determine the effect of the recrystallization of the BM-treated MCCs on their activity for producing H_2_ in photoreforming, the measured *r*H_2_ was plotted against *CrI*II-NMR (black symbols) and the amorphous proportion (red symbols) in the C4 region of the recrystallized MCCs (Figure 4). The *r*H_2_ increased gradually with an increase in *CrI*_II-NMR_, with a concomitant decrease in the amorphous proportion. Specifically, *r*H_2_ increased from 10.1 to 13.3 μmol h^–1^ when *CrI*II-NMR increased from 36.6% to 56.4%, and the proportion of the amorphous residues decreased from 63.1% to 43.6% (or the total recrystallinity of cellulose I and II increased from 36.9% to 56.4%). In addition, Figure 4 also shows that the correlation of *r*H_2_ with the amorphous proportion of the MCCs (*r*_A_^2^ = 0.93) was comparable to that with *CrI*_II-NMR_ (*r*_CrI_^2^ = 0.95). Therefore, on the basis of the earlier discussion, it is clear that the recrystallization of cellulose II in the BM-treated MCCs to water could account for the measured photoreforming activity.

Previous studies revealed that the reactivities of cellulose I/cellulose II mixtures after BM were distinct from that of amorphous cellulose for ethanolysis reactions,^62^ and the model for hydrolysis of mechanically decrystallized cellulose was revised to include recrystallization and hydrolysis of the cellulose I/II mixture. In this study, cellulose after BM (up to 2 h) contained both cellulose I and II in the MCCs (including both the as-prepared and water-exposed samples). The BM treatment caused a significant decrease in *DP* as well as conversion of cellulose I to amorphous cellulose, which underwent water-induced recrystallization to cellulose II, which was responsible for the increased *r*H_2_ in photoreforming. A previous study showed that cellulose II had relatively large lattice spacing compared to that of cellulose I, which benefited water uptake in the cellulose structure and hence improved the activity of photoreforming of cellulose for H_2_ production.^44^

### Formation of Cello-oligomers after the BM Treatment of the MCC

BM treatments resulted in the decomposition of the MCC structure to various extents, as discussed earlier. Hence, short chains of MCC (13 < *DP* < 162) and water-soluble (*DP* = 2–6) or partially soluble (*DP* = 7–13) cello-oligomers might be produced during the pretreatments.^70^ The cello-oligomers could affect photoreforming and contribute to H_2_ production because the water-soluble and/or partially soluble cello-oligomers could be more readily photoreformed than the insoluble parts of MCC. In this work, the treated MCCs were washed using water to obtain the filtrate, which was analyzed by HPLC-RI, in order to monitor the formation of soluble MCC fractions from the pretreatment processes. Figure S8 shows the peaks present in a blank sample corresponding to the RI signals of the mobile phase (*e.g.*, 5 mM H_2_SO_4_), along with the peaks of glucose, d-galactose, and formic acid in the control sample of the MCC-0 filtrate.

Water-soluble cello-oligomers were not detected in the filtrates of BM-0.5 and BM-2; however, these were shown to be present in the filtrates of BM-6, BM-16, and BM-24. In addition, anhydroglucose was also found in the filtrates of BM-16 and BM-24. Interestingly, these samples (i.e., BM-6, BM-16, and BM-24) also showed a significantly reduced *DP* (*i.e.*, 52.3, 31.4, and 32.1, respectively). The reduction in *DP* represented the breakage of cellulose chains, which led to the formation of water-soluble cello-oligomers (*i.e.*, cellodextrins, cellobiose, and anhydroglucose).

Small and soluble compounds from MCC degradation were more reactive than cellulose in photoreforming, such as glucose and formic acid.^1,19,71^ They were found in the filtrate of MCC-0 (*i.e.*, 6.4 × 10^–3^ g L^–1^ of glucose and 0.07 g L^–1^ of formic acid), and their concentration increased with the extension of BM treatment time, *i.e.*, from 0 to 24 h, the production of glucose increased to 7.1 × 10^–3^ g L^–1^ and formic acid increased to 0.11 g L^–1^. The production of these fractions via BM, however, was insignificant at <0.2% (on the basis of 20 g of MCC per L of water for preparing the filtrates) with comparison to the increase in *r*H_2_ for the photoreforming of BM-treated MCCs, *i.e.*, by ∼26.3% and ∼66.3% for BM-0.5 and BM-24, respectively. Their contributions to the enhanced H_2_ production were thought to be insignificant. To confirm the contribution of cello-oligomers from BM to the H_2_ production, a mass-balance experiment was conducted (Supporting Information), and the results are shown in Table S2. The mass loss in photoreforming MCC-0 was ∼0.031 g, which was similar to that in the photoreforming of BM-24 (0.034 g), suggesting that mass loss to the filtrate due to smaller compounds/cellulose oliogomers did not occur as a result of the BM pretreatment. The mass loss in this work could be due to the following reasons: (i) cellulose as the sacrificial agent being decomposed by consuming ·OH and (ii) mechanical losses during filtration and recovery. Therefore, combined with the conclusions, the contributions of water-soluble cello-oligomers as a result of the BM to the enhanced H_2_ production are thought to be insignificant.

A comparison of H_2_ production from cellulose photoreforming over Pt/TiO_2_ catalysts under similar reaction conditions (*i.e.*, in water, pH-neutral, at 20–60 °C) is shown in Table S3. The H_2_ production rate was normalized based on the amount of Pt loading because Pt was regarded as the active site of producing H_2_ via the reduction of protons in the photocatalysis system,^18^ and the normalized H_2_ production rate from the systems in this work is shown in Table S4. In this work, the normalized *r*H_2_ was improved (*i.e.*, from 66 600 to 110 800 μmol h^–1^ g_Pt_^–1^) after the 24 h BM pretreatment of the cellulose substrate, which was among the high-activity region in comparison with the state-of-the-art data (Table S3), with H_2_ production rates of 82 900–120 000 μmol h^–1^ g_Pt_^–1^.

The mechanism associated with the improvement in H_2_ production from photoreforming of the ball-milled cellulose is discussed below. BM pretreatment caused significant amorphization of cellulose, with completely amorphous cellulose formed after 6 h of milling. During the photoreforming process, amorphous cellulose in the ball-milled MCCs underwent a degree of recrystallization to cellulose II due to the exposure to water, the proportion of which increased with milling time, reaching *CrI*_II-NMR_ = 56.4% for BM-24. Under UV irradiation, active surface ·OH species on Pt/TiO_2_ are generated via the reaction between water and photogenerated holes (h^+^) on the catalyst. These ·OH species could then be transferred to the surface of cellulose. Compared to cellulose I, cellulose II has been shown to have high hydrolysis activity (Wada et al.^32^) with the cellulose II polymorph having increased uptake of water due to the larger lattice spacing. This together with the smaller particle size and shorter chain length of the milled samples increased the interaction of ·OH and cellulose II and, therefore, the hydrolysis rate. With hydrolysis of cellulose by the attack of ·OH proposed to be the first step of cellulose photoreforming,^44,52^ the changes in cellulose structure and particle size were proposed to enhance the (photo)hydrolysis of cellulose, forming intermediates such as sugars (and further oxidation products such as formic acid), which could hinder the recombination of photogenerated e^–^ and h^+^ on the catalyst more efficiently. As a result, the use of BM to alter the cellulose structure and improve its hydrolysis activity under photoreforming conditions increased the H_2_ production compared to that using the pristine cellulose. The promising improved energy efficiencies of BM at larger scale^36,38,40^ indicated that BM could be a potential pretreatment in the sustainable production of H_2_ from cellulose.

## Conclusions

Ball-milling (BM) physical treatment of microcrystalline cellulose (MCC) is effective to alter the physiochemical properties of MCC, which can benefit the photoreforming of MCC for H_2_ production. Herein, MCC was pretreated by BM to investigate the mechanism of how the pretreatment method affects the activity of the treated MCC in photoreforming. The findings show that the BM treatment could modify the MCC significantly with decreased particle size, *DP*, and *CrI*, especially after prolonged treatment time (>2 h). Importantly, amorphization of MCC was confirmed after the BM treatment, and the amorphous cellulose produced by BM went through recrystallization (to cellulose II) during photoreforming in the aqueous phase. On the basis of the analysis of the property and reactivity data, it was found that the proportion of recrystallized cellulose II in the BM-treated MCCs correlated well with the H_2_ production rate. The findings of this work indicate that recrystallization of amorphous cellulose to cellulose II, as well as the reduced MCC particle sizes after BM treatment, are responsible for the improved H_2_ production. This is proposed to be due to the more accessible structure compared to cellulose I, which improves the MCC–catalyst–media interactions.

## Supporting Information Available

Preparation method of the catalyst; schematic structure of cellulose; H_2_ production results of BM-treated MCCs; SEM and particle-size distribution of BM-treated MCCs; ATR-IR characterization and spectra of BM-treated and water-exposed MCCs; spectra and deconvolutions of ^13^C chemical shifts in the C4 region of MCCs from ssNMR characterization; HPLC analysis of the filtrates from washing of BM-treated MCCs; mass-balance experiment and its results from the photoreforming reaction of BM-treated MCCs; state-of-the-art of H_2_ production from cellulose photoreforming; and normalized *r*H_2_ of BM-treated MCCs (PDF)
