# Supplementary material for: Effect of Ball-Milling Pretreatment of Cellulose on Its Photoreforming for H2 Production
Source: ACS Sustain Chem Eng. 2022 Apr 4;10(15):4862–71. doi: 10.1021/acssuschemeng.1c07301 (PMC9098191; doi:10.1021/acssuschemeng.1c07301)
Supplement: Supplementary file 1 — sc1c07301_si_001.pdf [file sc1c07301_si_001.pdf]

Effect of ball-milling pre-treatment of cellulose on its photoreforming for H_2 production

Supporting Information (SI)

Lan Lan, Huanhao Chen, Daniel Lee, Shaojun Xu, Nathan Skillen, Aleksander Tedstone, Peter Robertson , Arthur Garforth, Helen Daly, Christopher Hardacre , Xiaolei Fan

Number of pages: 11

Number of figures: 8

Number of tables: 4

Contents:

1. Preparation method of 0.16%-Pt/m-TiO_2 catalyst.

2. **Fig. S1.** Schematic structure of cellulose

3**. Fig. S2.** Time course of H_2 production from photoreforming of MCC-0 and BM treated MCCs. Reaction conditions: 100 mg of substrate, 75 mg of 0.16 wt.% Pt/m-TiO_2 catalyst, 100 ml distilled water under the irradiation of UV-A lamp (incident photon rate: 62 μmol h^{-1} photons, 1 mol (6.0223×10^{23}) is 1 Einstein of photons.) at 40 °C for 5 hours.

4. **Fig. S3**. Distribution of the averaged H_2 production rate (rH_2, black scatters) and quantum yield (Φ_a, red scatters) from the photoreforming systems as a function of *DP* of the MCC-0 and BM-treated MCCs.

5. **Fig. S4**. SEM images of (a) MCC-0, (c) BM-2 (e) BM-16 (g) BM-24, and particle size distribution of (b) MCC-0, (d) BM-2 (f) BM-16 (h) BM-24.

6. ATR-IR characterisation of ball-milled MCCs

7. **Fig. S5**. ATR spectra of the MCC-0 (black spectrum), MCC-0.5 (red spectrum) and MCC-24 (blue spectrum).

8. Deconvolutions of ^{13}C chemical shifts in C4 region of MCCs from ss NMR charaterisation

9. **Fig. S6**. ^{13}C ssNMR spectra of (a) MCC-0, (b) BM-0.5-REC, (c) BM-2-REC, (d) BM-6-REC, and (e) BM-24-REC. Examples of deconvoluted ^{13}C ssNMR spectra for (f) crystalline cellulose I with amorphous cellulose in C4 region of MCC-0: crystalline residue α (I-α), residue β (I-β), residue γ (I-γ), para-crystalline residue δ (I-δ), and amorphous cellulose (A-α); and for (g) crystalline cellulose II with disordered cellulose in C4 region of BM-24-REC: crystalline residue a (II-a), residue b (II-b), residue c (II-c), residue d (II-d), amorphous a (A-a) and amorphous b (A-b).

10. **Table S1**. Crystalline index (*CrI*) calculated from ^{13}C ssNMR in C4 and C6 regions of cellulose I and cellulose II, and relative intensity of ^{13}C signal in glucose C1 region

11. ATR-IR characterisation of water-exposed BM-treated MCCs

12. **Fig. S7**. ATR-IR spectra of (a) MCC-0-WE, (b) recrystallised BM-treated MCCs: BM-0.5-REC (red) and BM-24-REC (blue), and (c) as-prepared BM-treated MCCs: BM-0.5 (red) and BM-24 (blue); (d) MCC-0 (grey) with grey spectra showing the change in 897 cm^{-1} band with milling time (BM-0.5 to BM-24), and (e) MCC-0-WE (black) with grey spectra showing the change in 897 cm^{-1} band with BM time after exposure to water (BM-0.5-REC to BM-24-REC).

13. **Fig. S8**. HPLC-RI analysis of the filtrates from MCC washing: (a) MCC-0 and BM-treated MCCs.

14. Mass-balance experiment

15. **Table S2.** Mass loss of the photoreforming reaction of MCC-0 and ball-milled cellulose

16. **Table S3.** The state-of-the-art of H_2 produced from cellulose photoreforming

17. **Table S4.** The normalised rH_2 (by Pt loading amount) of ball-milled MCCs

Preparation method of 0.16%-Pt/m-TiO_2 catalyst

0.16%-Pt/m-TiO_2 catalyst was prepared by wet impregnation. The procedure of making the 0.16%-Pt/m-TiO_2 catalyst is described as: 1 g m-TiO_2 support (mixed phase of rutile and anatase , Aldrich®) was dispersed in 9.58 ml of deionised water in a beaker and stirred for 10 min. Then 0.42 ml of the metal precursor solution (C = 0.01 g ml^{-1}, $H_2PtCl_6 \cdot 6H_2O$ dissolved in deionised water, Honeywell Fluka®) was added into the TiO_2 slurry. The mixture was stirred at 60 °C for 4 h, and the resulting catalyst was dried at 150 °C for 2 h and calcined in air at 500 °C for 2 h. Prior to the catalytic test, catalyst were reduced in pure H_2 at 200 °C for 30 min.

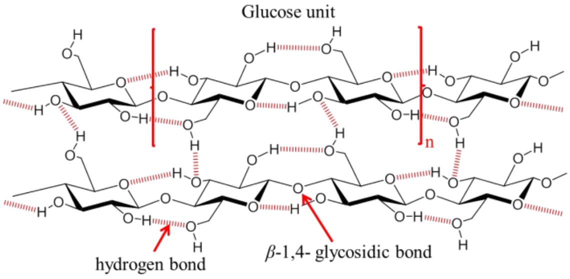

Fig. S1. Schematic structure of cellulose.

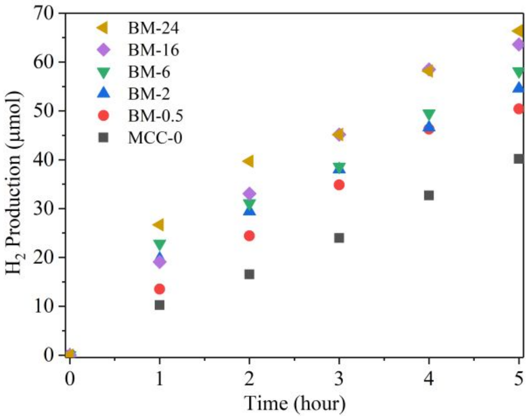

Fig. S2. Time course of H_2 production from photoreforming of MCC-0 and BM treated MCCs. Reaction conditions: 100 mg of substrate, 75 mg of 0.16 wt.% Pt/m-TiO_2 catalyst, 100 ml distilled water under the irradiation of UV-A lamp (incident photon rate: 62 μmol h^{-1} photons, 1 mol (6.0223×10^{23}) is 1 Einstein of photons.) at 40 °C for 5 hours.

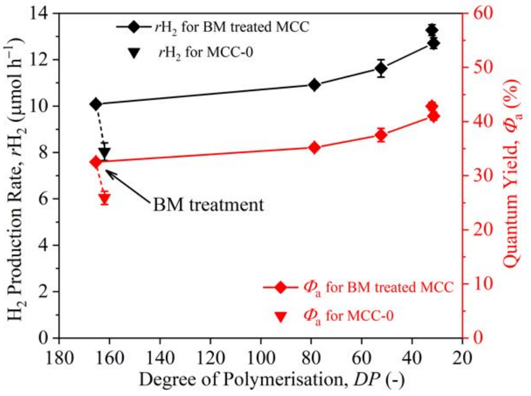

Fig. S3. Distribution of the averaged H_2 production rate (rH_2, black scatters) and quantum yield (Φ_a, red scatters) from the photoreforming systems as a function of *DP* of the MCC-0 and BM-treated MCCs.

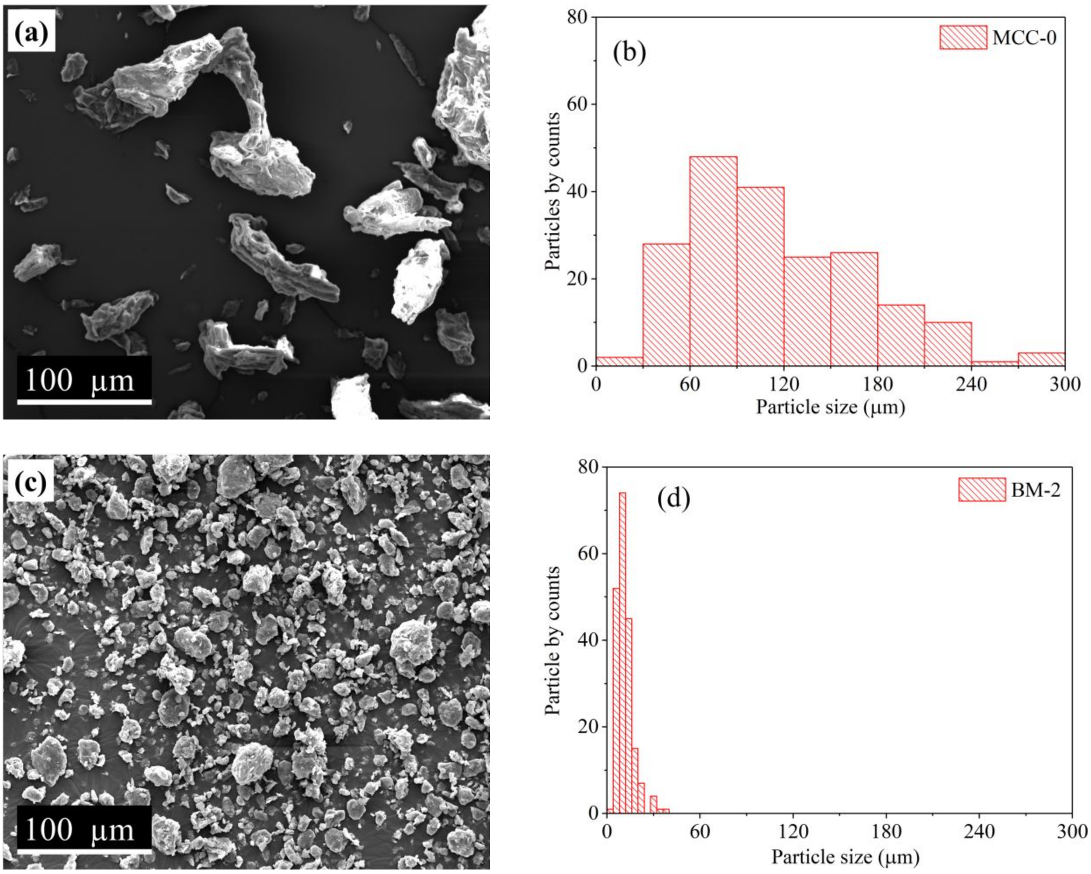

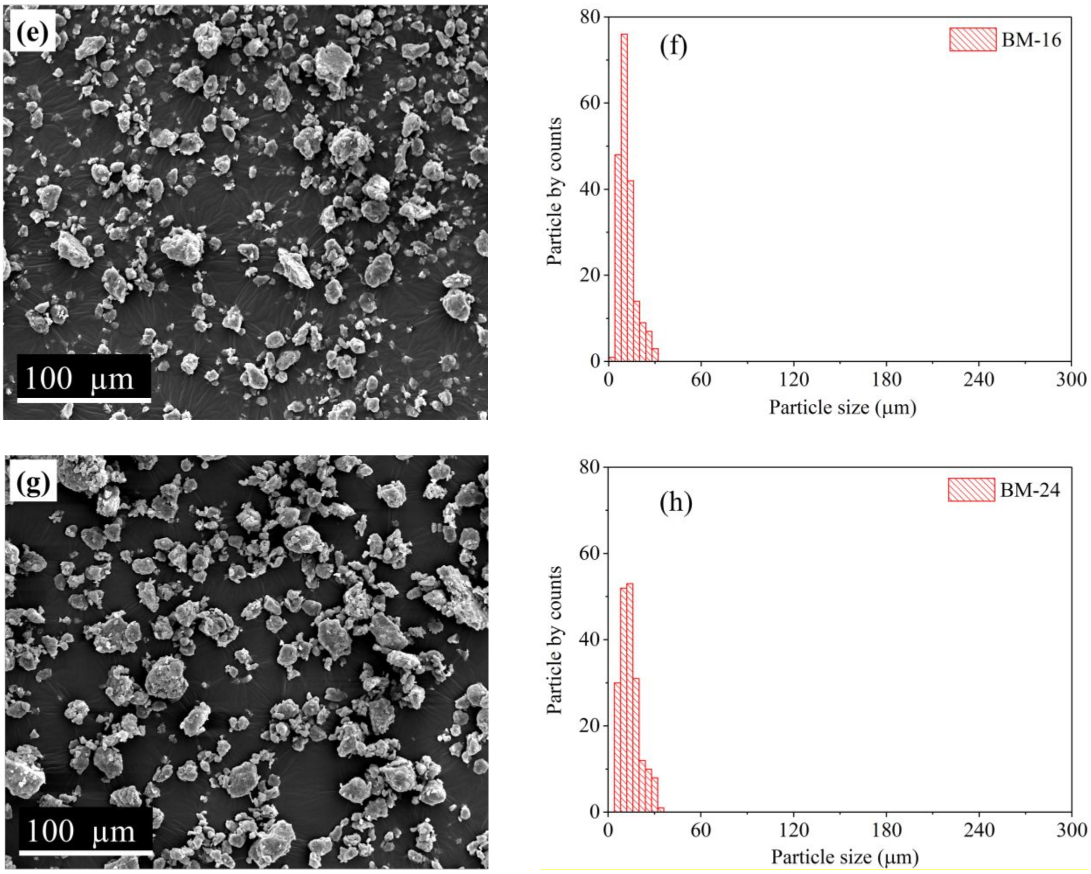

Fig. S4. SEM images of (a) MCC-0, (c) BM-2 (e) BM-16 (g) BM-24, and particle size distribution of (b) MCC-0, (d) BM-2 (f) BM-16 (h) BM-24.

ATR-IR characterisation of ball-milled MCCs

The conversion of cellulose I to amorphous cellulose after BM was also supported by ATR-IR characterisation as shown in **Fig. S5.** In detail, the disappearance of cellulose I IR absorption bands at 1106, 1315, 1335 and 1428 cm^{-1} in the BM-treated MCCs, and a band at 897 cm^{-1} representing amorphous cellulose were observed. Both XRD and ATR-IR characterisation showed the effectiveness BM for amorphising the MCC after ≥2 h treatment.

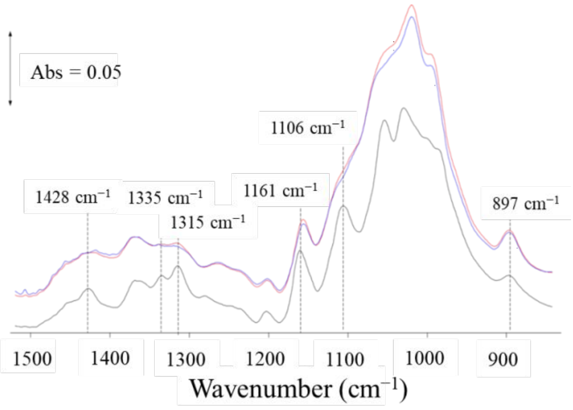

Fig. S5. ATR spectra of the MCC-0 (black spectrum), BM-0.5 (red spectrum) and BM-24 (blue spectrum).

Deconvolutions of ^{13}C chemical shifts in C4 region of MCCs from ss NMR charaterisation

In the C4 region of cellulose I (**Fig. S6f**), the ^{13}C chemical shifts were assigned to 4 crystalline signals (I-α: 89.4 ppm, I-β: 87.8 ppm, I-γ: 88.8 ppm, and I-δ: 88.4 ppm), and a broad signal for amorphous cellulose (A-α: 83.8 ppm).[1] While, in the C4 region of cellulose II (as shown in **Fig. S6g**), the ^{13}C chemical shifts were assigned to 4 crystalline signals (II-a: 87.5 ppm, II-b: 88.66 ppm, II-c: 86.3 ppm and II-d: 84.8 ppm), and 2 broad signals for amorphous cellulose (A-a: 82.85 ppm and A-b: 79.6 ppm).[2]

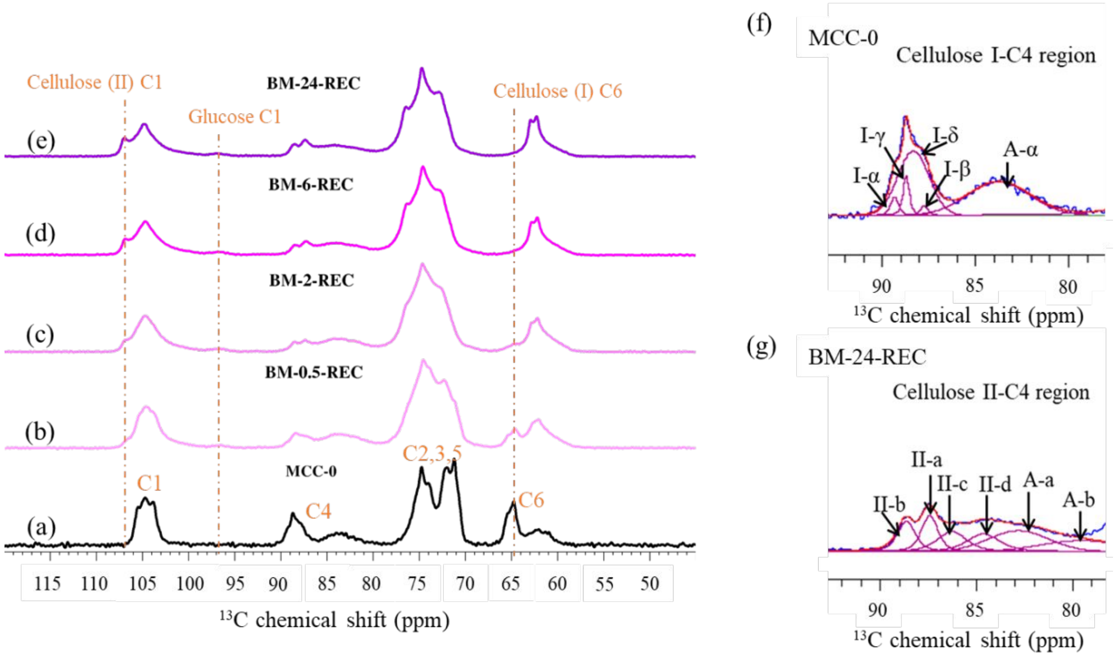

Fig. S6. ^{13}C ssNMR spectra of (a) MCC-0, (b) BM-0.5-REC, (c) BM-2-REC, (d) BM-6-REC, and (e) BM-24-REC. Examples of deconvoluted ^{13}C ssNMR spectra for (f) crystalline cellulose I with amorphous cellulose in C4 region of MCC-0: crystalline residue α (I-α), residue β (I-β), residue γ (I-γ), para-crystalline residue δ (I-δ), and amorphous cellulose (A-α); and for (g) crystalline cellulose II with disordered cellulose in C4 region of BM-24-REC: crystalline residue a (II-a), residue b (II-b), residue c (II-c), residue d (II-d), amorphous a (A-a) and amorphous b (A-b).

Table S1. Crystalline index (*CrI*) calculated from ^{13}C ssNMR in C4 and C6 regions of cellulose I and cellulose II, and relative intensity of ^{13}C signal in glucose C1 region

Sample	C4 region (%)[a]			C6 region (%)[a]			Glucose C1
	$CrI_{I\text{-}NMR}$	$CrI_{II\text{-}NMR}$	A[b]	$CrI_{I\text{-}NMR}$	$CrI_{II\text{-}NMR}$	A[b]	(%)[c]
MCC-0	55.6	0	44.4	54.6	0	45.4	0
BM-0.5-REC	57.6	36.6	63.1	46.7	28.2	68.0	3.8
BM-2-REC	47.8	41.3	55.5	25.5	38.1	63.1	5.4
BM-6-REC	0	53.3	46.7	0	48.7	51.3	5.5
BM-24-REC	0	56.4	43.6	0	57.5	42.6	5.5

[a] The total % can be > 100 %, because the relative weight of CrI_{I} and CrI_{II} is not taken into account here, *i.e.*, it should be $(1-x)CrI_{I}+(x)CrI_{II}+A$ ~100, where in the C4 region: x = 0, 62, 79, 100, 100, and in the C6 region: x = 0, 60, 79, 100, 100 for MCC-0, BM-0.5-REC, BM-2-REC, BM-6-REC and BM-24-REC, respectively; [b]the relative proportion of amorphous cellulose in C4 and C6 regions in the recrystallized BM-treated MCC, eq. (5) in the manuscript; [c]the relative proportion of the ^{13}C signal in glucose C1 region to the total amount of cellulose signals from the C1 region.

ATR-IR characterisation of water-exposed BM-treated MCCs

Recrystallisation of the BM-treated MCCs to cellulose II upon water exposure was also evidenced by ATR-IR and shown in **Fig. S7**). IR bands between 3230 and 3488 cm^{-1} (OH stretching region) assigned to inter and intra molecular H-bonding in cellulose[3, 4] changed significantly after BM (**Fig. S7 a–c**), which suggests the structural disruption of cellulose I. In addition, a band at □2918 cm^{-1} representing the formation of amorphous cellulose[5] was observed for the BM-treated MCCs, which was stronger for the as-prepared BM-treated MCCs (**Fig. S7c**) and weaker for the recrystallised counterparts (**Fig. S7b**). A shift from 2897 to 2876 cm^{-1} was also observed for the BM-treated MCCs (**Fig. S7b** and **S7c**), which suggests the conversion of cellulose I to amorphous/cellulose II.[6]

ATR spectra of the BM-treated MCCs show the band at 897 cm^{-1} corresponding to the emergence of amorphous cellulose. This band intensity is similar in all the IR spectra irrespective of BM treatment time (**Fig. S7**d) with only small differences in BM-0.5 spectra. However, for the recrystallised MCCs, there is a steady increase in the bands due to the formation of amorphous cellulose, and the shift to 894 cm^{-1} (as shown

in **Fig. S7**e) is reported for the formation of cellulose II.[4] The cellulose II content varies with ball-milling time, which shows no significant change between BM-16-REC and BM-24-REC.

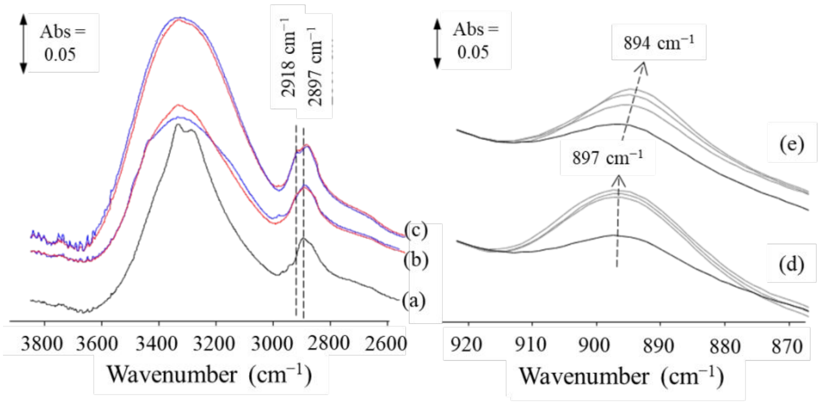

Fig. S7. ATR-IR spectra of (a) MCC-0-WE, (b) recrystallised BM-treated MCCs: BM-0.5-REC (red) and BM-24-REC (blue), and (c) as-prepared BM-treated MCCs: BM-0.5 (red) and BM-24 (blue); (d) MCC-0 (grey) with grey spectra showing the change in 897 cm^{-1} band with milling time (BM-0.5 to BM-24), and (e) MCC-0-WE (black) with grey spectra showing the change in 897 cm^{-1} band with BM time after exposure to water (BM-0.5-REC to BM-24-REC).

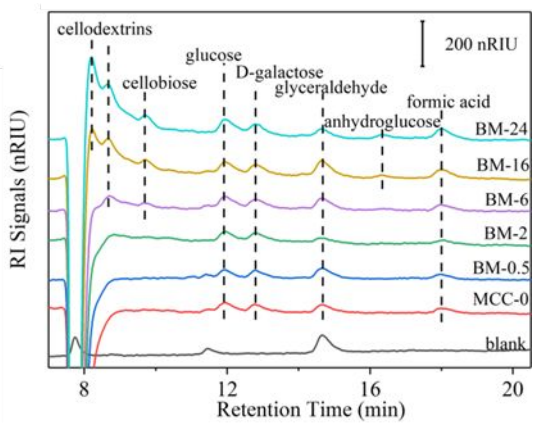

Fig. S8. HPLC-RI analysis of the filtrates from MCC washing: MCC-0 and BM-treated MCCs.

Experiment to check mass-balance

Additional experiments were performed to assess the mass-balance of the systems under investigation, that is, photoreforming of both pristine cellulose (MCC-0) and 24 h ball-milled MCC (BM-24) over 0.16 wt.% Pt/TiO_2 catalyst. The mass of the catalyst and cellulose (*i.e.,* MCC-0 and BM-24) were weighed before the reaction. After the photoreforming reaction, the mixture of cellulose and catalyst in the solution was

centrifuged (at 4400 rpm for 10 min) and filtered. The residue (mixture of cellulose and catalyst) was then dried at 100 °C for 24 h, and the dried residue was weighed as the total mass after reaction shown in **Table S2**.

Table S2 Mass loss of the photoreforming reaction of MCC-0 and ball-milled cellulose

Reaction feed			Total mass after reaction (g)	Mass loss (g)
Catalyst mass (g)	Cellulose	Mass (g)		
0.076	MCC-0	0.100	0.145	0.031
0.077	BM-24	0.101	0.144	0.034

Table S3 Comparison of H_2 production from cellulose photoreforming

Photocatalyst	H_2 production rate (μmol h^{-1} g_{Pt}^{-1})	Light source	Reference[a]
0.5 wt.% Pt/TiO_2	120000	Xe lamp	[7] Kondarides et al.
0.2 wt.% Pt/TiO_2	82900	UV-A lamp	[8] Lan et al.
0.5 wt.% Pt/TiO_2	45000	UV-A lamp	[9] Speltini et al.
1.0 wt.% Pt/TiO_2	23200	Xe lamp	[10] Caravaca et al.
0.5 wt.% Pt/TiO_2	8000	UV LED array	[11] Chang et al.
5.0 wt.% Pt/TiO_2	300	Xe lamp	[12] Kawai et al.

[a] references are listed below in the ESI.

Table S4 Normalised rH_2 (based on Pt loading) of the ball-milled MCCs

Cellulose sample	rH_2 (μmol h^{-1})	Normalised rH_2 (μmol h^{-1} g_{Pt}^{-1})
MCC-0	8.0	66600
BM-0.5	10.1	84100
BM-2	10.9	90800
BM-6	11.6	96600
BM-16	12.7	105900
BM-24	13.3	110800

References

1. Zuckerstätter, G.; Schild, G.; Wollboldt, P.; Röder, T.; Weber, H. K.; Sixta, H., The elucidation of cellulose supramolecular structure by 13C CP-MAS NMR. *Lenzinger Berichte* **2009,** *87*, 38-46.
2. Idstrom, A.; Schantz, S.; Sundberg, J.; Chmelka, B. F.; Gatenholm, P.; Nordstierna, L., (13)C NMR assignments of regenerated cellulose from solid-state 2D NMR spectroscopy. *Carbohydr. Polym.* **2016,** *151*, 480-487.
3. Kondo, T., The assignment of IR absorption bands due to free hydroxyl groups in cellulose. *Cellulose* **1997,** *4* (4), 281-292.
4. Oh, S. Y.; Yoo, D. I.; Shin, Y.; Kim, H. C.; Kim, H. Y.; Chung, Y. S.; Park, W. H.; Youk, J. H., Crystalline structure analysis of cellulose treated with sodium hydroxide and carbon dioxide by means of X-ray diffraction and FTIR spectroscopy. *Carbohydr. Res.* **2005,** *340* (15), 2376-91.
5. Mohan, T.; Spirk, S.; Kargl, R.; Doliška, A.; Vesel, A.; Salzmann, I.; Resel, R.; Ribitsch, V.; Stana-Kleinschek, K., Exploring the rearrangement of amorphous cellulose model thin films upon heat treatment. *Soft Matter* **2012,** *8* (38).
6. Schwanninger, M.; Rodrigues, J. C.; Pereira, H.; Hinterstoisser, B., Effects of short-time vibratory ball milling on the shape of FT-IR spectra of wood and cellulose. *Vib. Spectrosc* **2004,** *36* (1), 23-40.
7. Kondarides, D. I.; Daskalaki, V. M.; Patsoura, A.; Verykios, X. E., Hydrogen Production by Photo-Induced Reforming of Biomass Components and Derivatives at Ambient Conditions. *Catal. Lett.* **2007,** *122* (1-2), 26-32.
8. Lan, L.; Shao, Y.; Jiao, Y.; Zhang, R.; Hardacre, C.; Fan, X., Systematic study of H2 production from catalytic photoreforming of cellulose over Pt catalysts supported on TiO2. *Chin. J. Chem. Eng.* **2020**.*28*, 2084-2091.
9. Speltini, A.; Sturini, M.; Dondi, D.; Annovazzi, E.; Maraschi, F.; Caratto, V.; Profumo, A.; Buttafava, A., Sunlight-promoted photocatalytic hydrogen gas evolution from water-suspended cellulose: a systematic study. *Photochem. Photobiol. Sci.* **2014,** *13* (10), 1410-9.
10. Caravaca, A.; Jones, W.; Hardacre, C.; Bowker, M., H_2 production by the photocatalytic reforming of cellulose and raw biomass using Ni, Pd, Pt and Au on titania. *Proc. Math. Phys. Eng. Sci.* **2016,** *472* (2191), 20160054. https://doi.org/10.1098/rspa.2016.0054
11. Chang, C.; Skillen, N.; Nagarajan, S.; Ralphs, K.; Irvine, J. T. S.; Lawton, L.; Robertson, P. K. J., Using cellulose polymorphs for enhanced hydrogen production from photocatalytic reforming. *Sustain. Energ. Fuels.* **2019,** *3* (8), 1971-1975.
12. Kawai, T.; Sakata, T., Photocatalytic hydrogen production from water by the decomposition of poly-vinylchloride, protein, algae, dead insects, and excrement. *Chem. Lett.* **1981,** *10* (1), 81-84.
